# The Overall Survival, Complication-Free Survival, and Related Complications of Combined Tooth-Implant Fixed Partial Dentures: A Literature Review

**DOI:** 10.3390/dj4020015

**Published:** 2016-05-25

**Authors:** Peter Borg, James Puryer, Lisa McNally, Dominic O’Sullivan

**Affiliations:** 1 Regional Dental Clinic, Swieqi Road, Swieqi SWQ3410, Malta; peter.p.borg@gmail.com; 2 School of Oral and Dental Sciences, Bristol Dental Hospital, Lower Maudlin Street, Bristol BS1 2LY, UK; L.M.McNally@bristol.ac.uk (L.M.); D.J.OSullivan@bristol.ac.uk (D.O.)

**Keywords:** implant, survival, complications, fixed partial dentures

## Abstract

This paper reviews the literature regarding possible complications, complication-free survival, and overall survival of fixed dental prostheses that use both implants and natural teeth as abutments. The paper also provides clinical guidelines for treatment based on this literature review. An electronic search utilizing the MEDLINE, BIOSIS Citation Index, and Web of Science™ Core Collection databases was undertaken, and a review of the 25 selected texts studying 22 different patient cohorts was carried out. From a total of 1610 implants reviewed, 40 were lost (33 due to loss of integration and 7 due to fracture), whereas, out of a total of 1301 teeth, 38 were lost, of which 16 were due to fracture. Seventy-three cases of tooth intrusion were detected. From a total of 676 frameworks reviewed (metal *n* = 645, Zirconia *n* = 31), 7 fractured, while veneer material fracture occurred in 70 out of 672 bridges. Overall, 502 out of 531 tooth-implant fixed prostheses (TIPFs) remained functional, and 336 out of 439 prostheses showed no technical or biological complications and remained functional. Rigid TIFPs permanently cemented to teeth with sufficient coronal structure and with limited use of prosthetic attachments offer a good long-term treatment option to patients with good oral hygiene following sound implant placement. This mode of treatment should be used when free-standing implant-supported options may not be possible. Larger randomized control studies and other clinical studies comparing tooth-to-implant-connected treatment with other forms of treatment are needed to better understand the place of TIFP treatment in oral rehabilitation.

## 1. Introduction

Dental implants have originally been used with success in the treatment of edentulous patients [1,2], but more recently there has been a shift in their use towards the treatment of partially edentulous patients [3]. Implant treatment, including that of partially edentulous patients, has been proven to be successful, with favorable implant and implant-supported prosthesis survival rates [4,5]. One systematic review demonstrated an implant survival rate of 92–97% over a period of at least 5 years [4]. Similarly, a systematic review focusing on complications of implant-supported prostheses concluded that such dental prostheses had a survival rate of 96.4% after 5 years and 93.9% after 10 years [3]. Despite this, complications, both technical and biological, were frequent with up to 33.6% after 5 years [3].

Despite the positive results achieved with free-standing implant-supported fixed partial dentures (FPDs), certain clinical scenarios necessitate that root form implants be connected to natural teeth. Tooth-to-implant connection dates back to the early 1980’s [6], and the connection of teeth to implants as a desirable, rather than a necessary, option was first explored in 1986 by Ericsson *et al.*, who found that there was a satisfactory outcome with the use of osseointegrated titanium implants and teeth as abutments in the same fixed bridge reconstruction [7]. More recent studies have also demonstrated favorable results when connecting teeth and implants [8,9,10,11], with some studies showing similar treatment outcomes for tooth-to-implant and free-standing implant restorations [9].

**Topic debate:** The combination of natural teeth to dental implants was, and remains, a controversial topic in implant dentistry [12,13,14]. 

**Clinical relevance:** Clinical situations may arise where the connection of osseointegrated implants to teeth may be desirable. Such a scenario commonly arises in Kennedy Class I or II cases, where an implant may be connected to the terminal natural tooth via a three-unit bridge. Such a configuration would eliminate the need to place additional implants distal to the last natural tooth which, apart from increasing the cost, may be complicated by local factors such as reduced bone quantity or the presence of vital structures that would obstruct implant placement in that position. In such a case, avoiding complicated bone grafting procedures would decrease morbidity and the chance of complications. Such a technique may also preclude the need for cantilevered bridges supported by implants or teeth, which may not be desirable due to occlusal forces on the unsupported pontic.

The connection of teeth to implants can also be employed in long-span bridges involving many teeth and implants where strategically placed fixtures may be connected to the remaining teeth to restore the arch. In such cases, the use of teeth would simplify surgical treatment, reducing morbidity and cost while maintaining the periodontal ligament and corresponding proprioception from the natural teeth. Proprioception is particularly important in patients with bruxism, where the feedback from the remaining teeth may help to reduce stresses on the restoration [9,15]. In fact, it has been clinically demonstrated that there is an increased incidence of screw fracture and loosening as well as veneer fracture in free-standing implant-supported fixed prostheses (FSFPs) compared to tooth-implant fixed prostheses (TIPFs) [16]. Screw loosening is also more likely in bruxist patients in which the implants are overloaded with torsional force such as canines during guidance [6]. In these cases, the practitioner can reduce the susceptibility to torsional forces on the guiding tooth by splinting the implants to natural teeth as an anti-rotational measure. Preservation of the natural tooth may also aid in achieving a cosmetic result, which is often difficult to achieve with implant restorations, particularly when attempting to recreate the interdental papillae in the anterior region.

**Biomechanics and concerns:** The connection of implants to natural teeth presents a biomechanical challenge, due to the difference in mobility of the implant and tooth. A periodontally healthy tooth shows displacement values of around 28 µm in the vertical direction during physiologic function [17] and 100–120 µm in the horizontal direction when a force of approximately 5 N is obliquely applied to the crown, with posterior teeth exhibiting less horizontal movement than anterior teeth [18]. Conversely, osseointegrated implants show vertical displacement values of 2–3 µm under forces of 45 N [17], 12–66 µm in the labio-lingual direction [17], and 40–115 µm in the mesio-distal direction under a force of 20 N [19].

The differences in displacement values demonstrate that teeth move more readily within the periodontal ligament (PDL) than osseointegrated implants within bone. This difference in mobility led to dispute over the possibility of rigidly connecting natural teeth to implants, and it was recommended that non-rigid connectors (NRCs) were used instead, with claims that occlusal loads would be taken by the implant, with the abutment tooth acting as a cantilever if rigidly connected to each other [7,20,21,22]. It was postulated that such implant overload would consequently lead to peri-implant bone resorption, eventual failure of the implant, prosthetic problems [23], and possible hypofunction of the natural tooth resulting in disuse atrophy of the periodontal structures [22].

More recently, a number of authors believe that there is sufficient flexibility within the implant-restoration unit (without a NRC) to allow for movement of the tooth within the socket to a degree where support is also achieved from the tooth [24,25], resulting in a more equal force distribution between the tooth and the implant [26]. The sharing of force may be partly due to prosthesis and abutment screw flexibility, particularly in this case where gold abutment screws are used that are inherently less rigid than the more commonly used titanium screws, partly due to fixture movement in the bone and partly due to the flexibility of the rigid prosthesis. Such claims of equal force distribution have been demonstrated clinically by the strain-gauge analysis of loads applied to prostheses [27]. This reasoning could prove the use of any mobile elements in the prostheses unnecessary and support the use of rigid prostheses when connecting natural teeth to implants.

Simply described, a vertical bite force causes movement of the tooth within the periodontal ligament, resulting in a moment of force around the implant. The magnitude of this moment depends on the mobility of the tooth and implant, the length and flexibility of the prosthesis and prosthetic components, and the flexibility of the bone. If sufficient mobility is achieved in the implant-restoration unit, and the tooth is firm within the socket, then support for the fixed partial denture will be achieved from both the implant and tooth, and long-term complications can be averted. However, in cases where the implant-restoration complex is not flexible or the tooth is lacking support, it is unlikely that prosthesis will gain any support from the natural tooth, which would instead act as a cantilever, justifying the concerns of clinicians wishing to insert NRCs. It is worth noting that minimal movement at the abutment implant junction will translate to a larger translation of the tooth in the socket.

The following factors affect the biomechanics of the tooth-to-implant connections [28]:

- mobility of the natural tooth;
- number of teeth and implants to be connected;
- occlusal forces including:
  ○ magnitude,
  ○ duration,
  ○ distribution, and
  ○ direction;
- the force absorbing properties of the veneering material;
- rigidity of the prosthesis including length, thickness, and connectors (rigid or non-rigid);
- type of bone.

**Connection techniques:** When the connection of teeth and implants is required via a prosthesis, there are two main designs that may be considered: a prosthesis with either a rigid or a NRC. Despite the theoretical support for non-rigid connection, a number of studies have demonstrated an association between the use of NRCs and complications, most notably tooth intrusion [29,30,31]. Apart from deciding on the type of connector to use, the clinician must also consider where to position it, either on the implant crown or natural tooth crown, and whether to include stress-absorbing elements between the implant and prosthesis [32,33] as was available with the now discontinued IMZ Intramobile element (IME) system. The use of telescopic crowns or copings with locking screws in combination with rigid connectors [34,35] as well as cementation of the FPD with temporary cement for increased retrievability have been suggested.

**Clinical complications:** Along with the biomechanical problems that result from connecting natural teeth to implants, other issues may arise including problems related to the retrievability of the restoration, strain on the implant screw [36,37], framework and veneering material strain and fracture [36], tooth intrusion [38], and mechanical problems of the tooth.

(a) **Retrievability:** If a NRC is used with screw retention on the implant, then the implant portion of the reconstruction can easily be removed. If, on the other hand, a rigid construction is necessary, a one-piece casting will reduce retrievability because of the cement retention on the natural tooth abutment. To have a rigid restoration and maintain retrievability, a “screw-locked connector” may be installed in the restoration or a one-piece casting used with a telescopic crown on the natural tooth. This would be retained through the use of temporary cement or a locking screw.
(b) **Implant screw loosening:** The constant bending of the prosthesis caused by the disparity in implant and natural tooth mobility may result in the loosening of the screw or ultimately fatigue fracture [39].
(c) **Framework and veneer fracture:** Similarly, the constant bending of the restoration may lead to fracture of the framework and veneering material. The preference to keep the framework connectors narrow to increase the flexibility of framework must be balanced with the need for fracture resistance. The veneering material should also be flexible and preferably absorb some of the occlusal load without compromising material strength.
(d) **Tooth intrusion:** Possible reasons have been described, including diffuse atrophy, differential energy dissipation, mandibular flexure, fixed partial denture flexure, impaired rebound memory, debris impaction or microjamming, and the ratchet effect [38], yet the etiology for this phenomenon is not fully understood.
(e) **Tooth or implant fracture:** Conventional tooth-borne prostheses or FSFP fracture of the supporting tooth or fixture is a possibility [40], especially in cases where the tooth is heavily restored, and the root canal treated [31].

## 2. Aims and Objectives

### 2.1. Aims

To provide an overview of the literature regarding possible complications, complication-free survival and overall survival of fixed dental prostheses using both implants and natural teeth as abutments. The review will also strive to assess whether the literature currently available can provide sufficient data as to inform clinicians on how best to connect implants and teeth. If sufficient evidence is available, the aim is to then provide clinical guidelines for treatment in line with the available literature.

### 2.2. Objectives

- To assess which clinical situations are best suited for TIFPs.
- To determine overall survival and complication-free survival for TIFPs.
- To determine whether rigid or non-rigid connectors are the better option when connecting teeth to implants.
- To determine the potential of periodontally compromised and structurally compromised teeth to be used as abutments for TIFPs.
- To determine the best ways of attaching the framework to the natural teeth.
- To determine the ideal construction of TIFPs, including connectors, framework, and veneering.

## 3. Methodology

### 3.1. Search Strategy

An electronic search was performed via Web of Knowledge (Thomson Reuters TM) including the MEDLINE, BIOSIS Citation Index, Web of Science™ Core Collection databases in October 2014. Studies from 1984 to 2014 that were published in English were included in the search.

The following key words sequence was used to search titles:

TITLE: (tooth* or teeth* or partial*) AND TITLE: (implant* or implants* or fixture* or fixtures*) AND TITLE: (fixed partial denture* or prostheses* or prosthesis* or connected* or connection* or bridge* or partial*).

The search results were reviewed, initially by title, and then by abstract. Copies of the remaining studies were obtained and subjected to a full text review applying the inclusion and exclusion criteria in order to determine the final list of studies to be included in this review. The PICO (Participants, Intervention, Comparison, and Outcome) Question used to focus the literature search is described in Table 1.

### 3.2. Types of Studies Included

- Studies published in peer-reviewed journals in English;
- Studies with at least ten participants;
- Randomized control studies;
- Prospective cohort studies;
- Retrospective cohort studies;
- Clinical trials including prospective and retrospective, controlled or uncontrolled and multi-center studies;
- Only studies utilizing commercially pure titanium implants were included.

### 3.3. Types of Participants

- Patients requiring tooth-to-implant fixed prosthesis treatment;
- Partially edentulous adult male and female patients, including smokers;
- Patients with fixed or removable opposing dentitions;
- Patients free from active periodontal disease.

### 3.4. Types of Intervention

- Tooth-to-implant fixed prostheses treatment in the maxilla and mandible;
- Tooth-to-implant connection with rigid and non-rigid connectors;
- Tooth connection utilizing telescopic crowns;
- Applications of temporary and permanent cements in tooth-to-implant cases;
- Sectional and full-arch cases;
- Application of different framework and veneering material for tooth-to-implant prostheses;
- Treatment utilizing vital and RCT teeth.

### 3.5. Outcomes Recorded

- *Prosthetic complications* including: veneer/framework fracture, abutment/implant fracture, cement failure, screw loosening, and loss of prosthesis;
- *Biological complications* including: tooth intrusion, dental caries, dental periapical pathology, tooth fracture, tooth bone loss, dental mobility, fixture bone loss, plaque deposits, and bleeding on probing.

### 3.6. Types of Excluded Studies/Exclusion Criteria

- *in vitro* experiments;
- animal studies;
- case reports;
- literature reviews;
- studies making use of immediate implants or immediate loading of implants.

### 3.7. Study Selection

**Figure 1:**
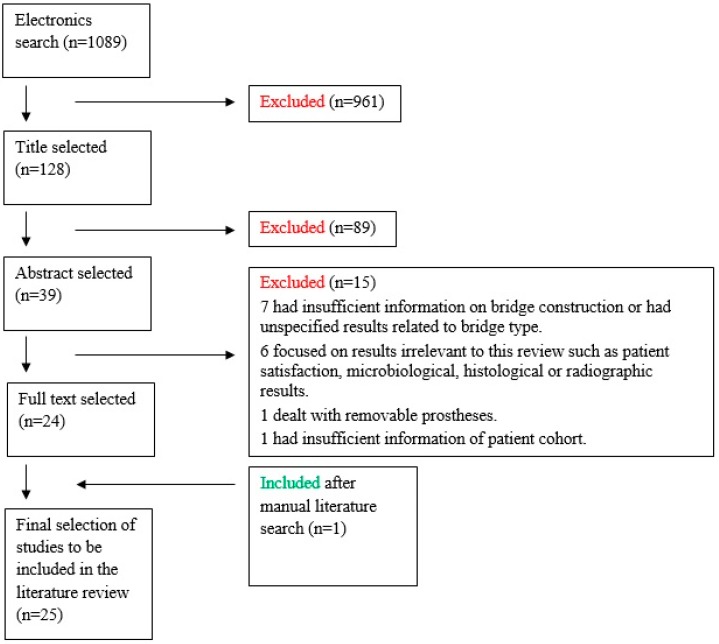
Flow chart of article selection.

The initial electronic search via Web of Knowledge (Thomson Reuters™) including the MEDLINE, BIOSIS Citation Index and Web of Science™ Core Collection databases returned 1089 articles. The title and abstract were used to reduce the number of relevant articles to 39. The full texts of these 39 studies were procured and read. Eight authors were contacted to clarify parts of eight different studies, of which only five authors replied. Of the three that did not reply, one article was excluded, while the other two were retained. After applying the inclusion and exclusion criteria, 25 articles were selected for the literature review (Figure 1).

### 3.8. Study Validity and Clinical Relevance

A number of the studies identified through the abstract dealt with implant rehabilitation as a whole, combining results for FSFPs and TIFPs together. These studies were not considered for this literature review. Further studies were excluded either because they failed to sufficiently describe the construction of the bridge or because the measured outcomes were not relevant to this review.

The 25 studies examined differed in their aims, methodologies, and data collection using several measures to assess TIFPs. Care was given in selecting the final studies, giving consideration to their clinical relevance and number of extraneous variables that would make it difficult in relating the causative factor to the obtained results.

The study outcomes noted can be broadly divided into two types:

*Biological*—related mainly to marginal bone levels around teeth and implants as well as to tooth intrusion and natural tooth condition including caries, periapical pathology, mobility and fracture, plaque accumulation, and gingival status.

*Prosthetic*—related to the implant and prosthesis including veneering material and framework condition, implant and implant abutment status, cement integrity, and implant screw loosening.

### 3.9. Data Extraction and Analysis

Each of the 25 included studies had their data extracted and inputted into a spreadsheet (Appendix 1) purposely designed for this study by a single reviewer. The spreadsheet included fields for the following data:

- study title, author names, publishing journal, and year of publication;
- institution where the study was conducted;
- type of study design;
- study aim;
- number of participants;
- number and type of prostheses, whether implant or tooth and implant-supported;
- bridge constructions;
- the periodontal condition of the teeth involved;
- the quality of the opposing dentition;
- method of prosthesis fixation to the supporting teeth and implants;
- observation period;
- biological and prosthetic complications.

Table 2 presents a list of all the studies selected for data extraction and analysis. Four of the studies followed the same cohort of patients over time [41,42,43,44]. Although all of the included studies were comparable, not all of the studies included answered all the questions asked by this critical review.

When it came to calculating overall prosthesis survival and complication-free survival, the prosthesis was used as the unit of measure. For the purpose of this study, *overall survival* refers to any prosthesis that remained functional in the mouth despite any complications that did not require remaking the restoration, and *complication-free survival* refers to prosthesis survival without any need for further treatment by the practitioner.

### 3.10. Study Quality Assessment and Risk of Bias

An assessment checklist based on that presented by Downs and Black in 1998 [45] was completed for each article in order to assess the quality of the study in relation to:

- *Reporting*;
- *External validity*—addressing the relevance of the findings to the general population;
- *Bias*—addressing bias in measuring the intervention and the measurement of the outcome;
- *Selection bias*—which addresses bias in subject selection;
- *Power*—attempting to determine whether the negative findings may be due to chance.

Each of the assessment questions were answered with a “Yes” contributing 1 point to the quality score, “No” not contributing to the quality score, or “Unable to determine” also not contributing to the score. Two criteria in the checklist had a variable scale, one from 0 to 2 and one from 0 to 5.

## 4. Results

Twenty-five texts studying combined TIFPs in 22 different patient cohorts were selected for analysis. The majority of studies dealt with a mixture of short- and long-span bridges. However, nine dealt with solely short-span restorations (<5 units) and one dealt only with long-span bridges (>5 units). Twenty-one of the studies were carried out in institutions such as dental hospitals, dental schools, and specialized clinics, another two were multi-center studies, and two were carried out in private clinics. The studies were carried out by specialists, university professors, and general dental practitioners. Of the selected texts, 13 described prospective cohort studies (four of which followed one patient cohort) and another 12 described retrospective cohort studies. Five studies used cross-arch controls, two of which had the treatment assigned to each side randomly. In six studies, the TIFP treatment was assessed against a group of FSFPs. The studies dealt with commercially available implant systems and were carried out over an observational period of at least 2 years, and up to 15 years in one study. In five of the studies, some patients were observed for less than 1 year.

**Figure 2:**
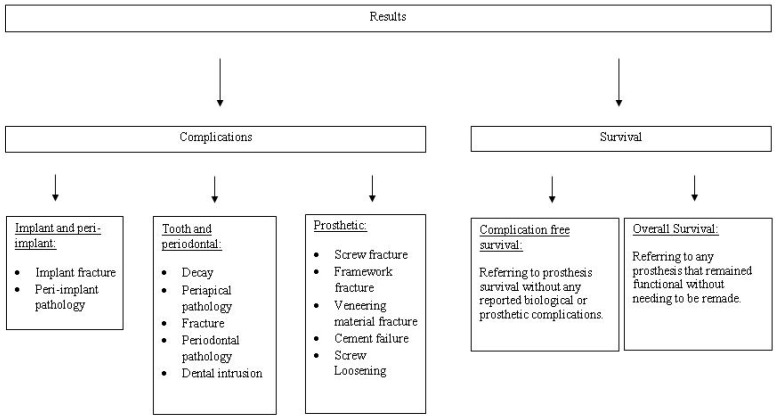
A flow diagram of outcomes assessed in this analysis.

The results of this review are divided into two groups: one dealing with complications and the other dealing with survival rates. Complications relating to tooth and implant rehabilitation are further divided into three sections. One section describes complications associated with the implants, another section describes complications associated with the abutment teeth, and the third section describes complications associated with the restoration itself. Similarly, all the studies that gave data on overall survival and complication-free survival are included in the survival section (Figure 2).

### 4.1. Complications

#### 4.1.1. Implant and Peri-Implant Complications

Of the 25 selected studies, 23 went into detail on implant related complications in TIFPs (Table 3). The reviewed studies dealt with at least thirteen different implant brands placed both in the maxilla and mandible supporting short- and long-span TIFPs made out of veneered metal. In one study, veneered zirconia bridges were used. The implants supported a mixture of rigid and non-rigid constructions that were either screw-retained or cemented via temporary, semi-permanent or permanent cements onto the implants. Out of a total of 1610 implants connected to teeth, 40 implants were lost over observation periods that ranged from 6 to 180 months. The majority of implants lost were as a result of a loss of integration, either due to peri-implant disease or primary biological complications that eventually resulted in mobility (*n* = 33). The remaining implant losses occurred due to fracture (*n* = 7) (Table 4).

Twenty-one of the studies reviewed also went into detail on peri-implant bone changes, which are summarized in Table 3. In some of the studies reviewed, significantly more bone loss was observed around implants supporting FSFPs than those supporting TIFPs [9,29,40]. Similarly, a study by Palmer *et al.*, which made use of 19 implants, connected to teeth in three-unit TIFPs, in Kennedy Class II cases, showed no significant bone loss around implants connected to teeth but instead noted 10 cases where there was a gain in peri-implant bone levels. In the other nine implants, bone loss of up to 1.2 mm was detected over 36 months [8]. Four texts [41,42,43,44] following the same cohort of 23 patients over 10 years also found less bone loss around TIFP implants than around FSFP implants. Significantly less bone loss was detected at the 2nd and 10th year around implants connected to teeth than around the implants in the cross-arch control group [41,44]. In this study, marginal bone loss was minimal, with 0.5–0.7 mm lost over 10 years in connected implants.

Unlike the above mentioned studies, in a retrospective study by Naert *et al.* including 339 tooth connected implants, there were no statistically different bone level changes from 0 to 6 months between the implants supporting FSFPs and those supporting non-rigid and rigid TIFPs. There was also no statistical difference in bone loss from 6 months to 15 years between the free-standing group (0.02 mm/year) and the non-rigid group (0.04 mm/year). There was, however, a statistically significant difference between the rigid group (0.09 mm/year) and the free-standing group (*p* = 0.004) [30]. In another retrospective study by Naert *et al.,* thirty-one implants were connected non-rigidly, and 41 were connected rigidly to teeth; no statistical difference was detected between bone loss around distal implant in FSFP and TIFP cases [46]. Rigid and non-rigid bridge constructions were also assessed in a study by Block *et al.*, which compared three-unit, non-rigid and rigid TIFPs. Four implants developed bone loss greater than 2 mm over 60 months. In this study, there was no significant difference in bone loss around rigidly and non-rigidly connected implants, with an average of 0.91 mm of bone loss over the 5-year study period [31]. Similarly, in a study involving 41 implants in long-span rigid and non-rigid cases, bone loss of less than 1 mm was reported in all connected implants, except in two patients where three fixtures demonstrated bone loss exceeding 1 mm but no more than 3 mm [7].

Long-span bridges (>5 units) were also studied in another retrospective study involving 112 implants that were rigidly or non-rigidly connected to teeth. In this study, 46 implants showed marginal bone loss after a year, 31 of which had up to one thread, 11 had up to two threads, 2 had up to three threads, and 2 had up to four threads of bone loss. Over the next four years, the progression of marginal bone loss was minimal. In this study, six implants were lost due to loss of integration [47]. A retrospective study by Cordaro *et al.* also followed long-span TIFPs rigidly and non-rigidly connecting implants to teeth with healthy and compromised periodontal ligaments. This study gave more positive results, with 87 out of 90 implants showing stable bone levels during the observation period while 3 had bone loss greater than 2 mm [34].

A study by Koczorowski and Surdacka comparing bone loss for 76 posterior implants placed in one or two stage procedures supporting TIFPs also gave positive results, with values for marginal bone loss in line with guidelines set by Albrektsson for implant success [48]. The mean bone loss at the implants after 2 years of using fixed partial dentures supported on mixed abutments was 0.70 mm ±0.50 and 1.73 mm ±0.41 after 6 years. This study showed no significant difference in marginal bone loss around implants placed in one or two stage procedures and connected to teeth [49]. On the other hand, in another study, 185 implants were connected to 220 teeth in six centers and described by Lindh *et al.* Seventy-four implants were followed for 3 years during which there was a statistically significant loss of bone over the first year in both jaws (*p* < 0.01) (maxilla: 0.33 mm, standard deviation (SD) 0.56 mm; mandible: 0.36 mm, SD 0.49 mm), with 9 out of the 74 implants losing more than 1 mm of marginal bone during the first year. Changes in marginal bone level during the second and third years did not reach significant levels (*n* = 9: 0.3 mm, SD 0.9 mm; *n* = 65: 0.1 mm, SD 0.5 mm) [50].

A long-term study by Bragger *et al.* followed partially edentulous patients restored with SCs, FSFPs, and TIFPs over 10 years. Twenty-one patients had 22 implants rigidly connected to teeth supporting 22 TIFPs. Although no measurements for bone loss were given, three implants were reported lost, and a further three implants needed treatment for peri-implantitis. Statistically significantly fewer biological failures occurred with FSFPs compared to TIFPs (P 1/4 0.022) [51]. Comparisons can be drawn with the study by Naert *et al.*, where rigidly connected implants demonstrated significantly more bone loss than implants supporting FSFPs. Similarly, in a prospective study by Tangerud *et al.*, 85 abutments were rigidly connected to teeth through long- and short-span bridges. Two of these implants were lost after prosthetic loading, and a bone reduction of 0.8 mm ± 1.1 mm was detected around implants from the time of loading to the 3-year review [52].

A study by Ozkan *et al.* also examined implants supporting SCs, FSFPs, and TIFPs. Following loading, the nine implants connected to teeth met the success criteria for mobility, and all implants were surrounded by stable healthy tissue with good hygiene. There were no significant differences in crestal bone level changes between TIFP and FSFP implants [53]. Likewise, Romeo *et al.* studied the performance of implants supporting SCs, TIFPs, and FSFPs. Although no measurements for marginal bone loss were given, it is worth noting that, in this prospective study, 13 TIFPs were supported by 31 implants of which 90.6% survived, compared to 96.1% for FSFPs. Implant success was 70.6% for TIFPs, compared to 73.8% for implants supporting FSFPs. Survival rates for implants supporting TIFPs and FSFPs were similar [54].

One study by Nickenig *et al.* retrospectively analyzed 142 implants connected to 132 abutment teeth in 84 TIFP implants (rigid *n* = 56, non-rigid *n* = 28), of which 40% were three-unit, and 33% were of five or more units. In this study, no implants needed to be removed, and less than 1% had probing depths of more than 5 mm after 5 years [35].

#### 4.1.2. Natural Tooth and Periodontal Complications

Nineteen of the 25 studies selected for this review went into detail about the condition of the natural tooth abutment supporting TIFPs (Table 5). In these studies, teeth were either rigidly or non-rigidly connected to implants via long- or short-span bridges. In all but one study, teeth were permanently or temporarily cemented to the prostheses either directly or indirectly through a coping; otherwise, the prosthesis was left uncemented with the tooth functioning as a telescopic abutment for the prosthesis. In one study, the prostheses were not cemented, but instead screwed onto a coping fabricated with a thread.

The selected studies used teeth with healthy periodontia showing varying degrees of bone loss around them. In none of the studies did the teeth show more than physiological mobility. Two studies used only vital teeth as abutments, seven studies used both RCT and vital teeth, and the other nine studies failed to give information on the endodontic status of the abutment teeth prior to treatment. The RCT teeth were restored with post and cores. In the studies reviewed, 38 out of a total of 1301 teeth were lost over observation periods, varying from 6 to 180 months. Of the 16 teeth lost to fracture, at least 6 were previously RCT. Unfortunately, of the studies that observed tooth fracture, only two specified whether the fractured teeth were previously RCT [31,52], and it is not known if the other 10 fractured teeth were also RCT. Of the 1301 teeth reviewed, 73 teeth intruded, 35 of which were detected in one study that used photographs to assess intrusion. Of the 73 intrusions, 51 occurred in non-rigidly connected teeth, 8 in rigid bridges with failure of the cement or breakage/loosening of the connector locking screw, and 14 were observed in rigidly connected teeth with no documented failure of the cement. In some of these 14 cases, temporary cement was used to fix the prosthesis to the natural abutment.

Out of 34 teeth used as abutments in a study by Akca and Cehreli, one tooth needed to be root treated, although none were lost during the observation period. In this study, only four of the teeth used as abutments were previously root canal treated, and none of the teeth used had fractured. There were also no cases of intrusion documented. It is worth noting that the teeth were permanently cemented to the rigid prostheses in all cases [9]. The absence of tooth intrusion was contrary to what was observed in the study by Block *et al.*, where rigid bridges were cemented with temporary cement and petroleum jelly, resulting in a 44% intrusion rate of rigidly connected teeth, 12.5% of which showed intrusion of more than 0.5 mm. Although a significant amount of rigidly connected teeth did intrude, this was still less than the 66% of non-rigidly connected teeth that intruded [31]. In the study by Block *et al.*, RCT teeth were a weak point, with all five fractures occurring in endodontically treated teeth with posts (27 of 60 teeth used were RCT). Two fractures occurred in rigidly connected teeth, and three in those non-rigidly connected. In this study, there was significantly more bone loss around rigidly connected teeth than non-rigidly connected teeth [31]. A study by Hosny *et al.* connected 30 teeth, mostly with rigid connectors to implants. Sixteen of the teeth were temporarily cemented. Despite this, no cement failures were detected, and no teeth intruded. In this study, one tooth developed a periapical lesion 6 months after connection [29].

Similarly, a study of 19 vital teeth by Palmer *et al.* showed no cases of intrusion even though the rigid frameworks were only temporarily cemented to natural teeth. It is interesting to note that the TIFPs were also temporarily cemented to the implant abutment; thus, when debonding occurred on one abutment, it subsequently occurred on the second abutment, most likely preventing intrusion from occurring. No significant changes in bone level were reported around teeth in this study, and no fractures were observed [8]. Cases of intrusion were also not observed in a study by Heinemann *et al.*, where rigid TIFPs were temporarily cemented to teeth. However, in this study, two teeth needed to be extracted and one endodontically treated [55]. A study by Mundt *et al.* observed 40 teeth rigidly connected to implants with semi-permanent cement. In this study, no teeth suffered complications, and there were no signs of intrusion. It is worth noting that, in this study, as was the case in the study by Heinemann *et al.*, the author made use of permanently cemented copings to protect the teeth before cementation of the finished prosthesis [56]. Similar copings were not used in a long-term study by Bragger *et al.*, where 24 natural abutments were rigidly connected to teeth. In this study, four teeth were lost due to caries following loss of retention from the natural abutment despite being permanently cemented. In a number of cases, the bridge was cemented to the implant abutment and could not be removed despite having debonded from the tooth. Most of the lost tooth abutments were previously RCT and restored with posts [51].

In a study by Lindh *et al.,* 26 Kennedy Class I patients had TIFPs installed on one side of their maxilla, and FSFPs on the contra-lateral side. Twenty-six teeth were connected, of which 15 were endodontically treated, 20 of the natural abutments were canines, 3 premolars, and 3 incisors. In this text, only one tooth fractured after more than 2 years of function. The low fracture rate, despite the number of RCT teeth, as compared to the study of Block *et al.*, may be due to the large number of canines used as abutments. In this study, no tooth mobility and no intrusions were detected, though three teeth devitalized [40].

A study by Ericsson *et al.* included 29 teeth rigidly or non-rigidly connected to 40 implants in 11 long-span TIFPs. One case of intrusion from a tooth supporting a non-rigid bridge was noted. No loss of alveolar bone was reported around natural abutments throughout the duration of the study, and, apart from the intruded tooth, no other complications were reported [7]. A retrospective study by Cordado *et al.,* which also followed long-span TIFPs rigidly and non-rigidly connected to teeth with healthy and periodontally compromised teeth, observed four cases of intrusion out of a total of 72 connected teeth. Intrusions occurred only in non-rigidly connected teeth and, interestingly, in teeth with healthy periodontia. No cases of intrusion were evident in teeth with reduced periodontal support, suggesting no link between the amount of periodontal support and the likelihood of intrusion. In this study, no teeth developed carious lesions, fractures, or periodontal pathology [35].

In a separate retrospective study, 85 teeth were connected to implants in long- and short-span TIFPs. Five teeth were lost due to fracture and endodontic complications. The text also reported no marginal bone loss around teeth after 1 year. There was, however, “minor” bone loss for the 80 teeth reviewed at 3 years, and 2 mm bone loss around 3 teeth out of the 47 reviewed at 5 years. Three cases of intrusion were detected—one occurred in a case with non-rigid tooth-to-implant connection, and two where non-secured telescopic crowns were used [47]. In a second study by Lindh *et al.*, 220 teeth were connected either rigidly or non-rigidly to implants. Over a three-year observation period, although no teeth were lost, one tooth required endodontic treatment and two teeth developed caries. In this study, 11 teeth showed signs of intrusion. Eight of these teeth were related to prostheses supported by one implant and one tooth. In all cases, the intruded teeth were non-rigidly connected to implants (three with telescopic crowns, four with non-rigid attachments, *i.e.*, without locking screws) or with rigid connectors where the locking screws had fractured (*n* = 2) or loosened (*n* = 2) [50]. On the contrary, four texts followed the same cohort of 23 patients over 10 years, where teeth were permanently cemented to rigid prostheses. In this study, no cases of intrusion were described. However, one tooth was lost due to caries and endodontic problems, which were detected at the ten-year follow up. Only one tooth had more than physiological mobility after 10 years [41,42,43,44].

Naert *et al.* connected 313 teeth to implants in 140 TIFPs, of which 34 were of a non-rigid construction, 49 rigid, and 57 mixed. Nineteen teeth, incorporated in nine separate prostheses, intruded. All of the intruded teeth suffered cement failure and debonded from the prosthesis. Two teeth reviewed in this study fractured, 3 teeth needed extraction due to decay or periodontal problems, and 11 teeth in nine prostheses had periapical pathology [30].

In a 36-month prospective study by Tangerud, 86 teeth, 40 of which were RCT and restored with posts, were rigidly connected to implants. One RCT canine had to be extracted due to fracture, while two teeth developed pockets greater than 5 mm, and one developed a draining sinus. Pockets of 4 mm or more were found at 22% of teeth at Year 1 and 19% at Year 3, while bone reduction was of 0.1 mm ± 0.8 mm from the time of loading to the 3-year review [52].

In a study by Nickenig *et al.*, 132 abutment teeth were rigidly (*n* = 56) and non-rigidly (*n* = 28) connected to 142 implants in bridges, of which 40% were three-unit, and 33% five or more unit. During the observation period, three teeth were lost because of periodontal inflammation or periapical abscess. After 5 years, as many as 8% of abutment teeth required periodontal treatment, while only three needed restoring and one needed root canal therapy [35].

#### 4.1.3. Prosthetic Complications

Twenty texts went into detail regarding the prosthetic complications that arose in 17 patient cohorts over an observation period that varied from 12 to 180 months (Table 6). Out of 676 frameworks reviewed (metal *n* = 645, zirconia *n* = 31), 7 frameworks fractured. Two fractures occurred in rigid zirconia bridges, and five in metal constructions, with two of these fractures occurring around prosthetic attachments. Veneer material fracture was described in 70 cases out of 672 bridges. Bridges were constructed with porcelain (*n* = 558), acrylic (*n* = 54), or composite (*n* = 38) veneering. It is worth noting that 10 veneer fractures occurred in the one study where zirconia frameworks (*n* = 31) were used. Three studies failed to describe the material in which veneer fractures occurred. However, out of the studies that did, ceramic veneer fracture occurred in 46 of 391 TIFPs, while acrylic and composite fracture occurred in 9 of 50 TIFPs. Fifteen fractures in three studies [47,50,52] were not attributed to any material. Despite this, in the study by Lindh *et al.,* the author wrote that “prostheses using metal-ceramic superstructures showed less wear, fewer technical complications, and a higher level of long-lasting esthetic results compared with superstructures using gold-alloy framework and acrylic resin veneers.” A similar conclusion was reached in the study by Kindberg *et al.*, which dealt mostly with rigid constructions. This text concluded that prostheses using metal-ceramic superstructures resulted in fewer technical complications, less wear, and longer lasting esthetic results compared to superstructures using gold-alloy framework and acrylic resin veneering. In this study, equal numbers of acrylic and porcelain veneered prostheses were used [47].

In a study by Cordaro *et al.*, which made use of 15 metal-ceramic and 4 metal-composite prostheses, one prosthesis had damage to the composite veneer, which was replaced with porcelain over the existing metal framework. This patient’s opposing dentition was made out of a fixed prostheses [34]. Veneer fractures were a common occurrence in a short-term retrospective study by Rammelsberg *et al.,* who observed chipping in seven metal-ceramic prostheses [11], and in a study by Heinemann *et al.,* which exhibited five veneer fractures from a total of 65 metal-ceramic prostheses [55]. In a study by Noda *et al.*, 136 metal-ceramic TIFPs were compared to 13 metal-ceramic FSFPs. In the connected group, there were 22 veneer fractures, compared to 7 fractures in the non-connected group [39]. A study by Lindh *et al.* using metal-ceramic prostheses did not report any veneer fractures although two temporary cement failures were detected. This study also reported one implant screw fracture compared to three abutment screw fractures in the FSFP group [40].

In the studies reviewed, 18 implant screws, including abutment and prosthesis screws, loosened. Cement failures were evident in 132 abutments, 17 where permanent or semi-permanent cement was used, and 90 were temporary cement was used (80 of which occurred in one study). In 25 cement failures, it was not specified which cement was used. Of the studies reviewed, only one study used temporary cement alone, one study used semi-permanent cement, four studies used permanent cement, and six studies used a number of different cements. The other studies reviewed failed to describe which cement was used.

The importance of cement selection was outlined in a study by Bragger *et al.* In this study, four cases of cement failure led to loss of the TIFPs due to resultant caries of the abutment teeth. The use of permanent cement on the implant abutment made it difficult to retrieve the prosthesis once debonded from the natural abutment, resulting in decay. In this study, two TIFPs had fracture of the porcelain veneer, which was not sufficient to warrant a remake [51]. The opposite was evident in a study by Palmer *et al.* In this study, temporary cement was used to secure the prosthesis to the natural tooth and implant abutments. As a result, when bridge decementation occurred in eight subjects, in all except one, decementation happened on both the tooth and implant. In this study, eight bridges displayed chipping of the composite veneering material, which occluded against fixed opposing teeth. None of the abutment teeth developed carious lesions [8].

Four studies reported no prosthetic complications. In these studies, 70 of 74 bridges were metal-ceramic, and 4 of 74 were metal-acrylic. The prostheses used were both of a rigid and non-rigid construction and permanently cemented on 57 natural abutments, temporarily cemented on 16 natural abutments, while no indication was given for 13 abutments. The TIFPs were cemented on some implant abutments and screw-retained on others [9,29,53,54]. One long-term study following 23 metal-acrylic TIFPs over 10 years reported few prosthetic complications. In this study, the test group was compared to a control group of 23 FSFPs. The only prosthetic complication related to three loose abutment screws, as compared to two loose abutment screws in the FSFP group [41,42,43,44].

As previously described, framework fracture occurred in three studies. In one study, zirconia frameworks were used. This study showed two framework fractures, both in patients with parafunctional habits. Ten veneer fractures also occurred in patients with parafunctional habits [56]. Three metal framework fractures occurred in a study by Naert *et al.* This study also showed 25 cement failures and three abutment screw fractures in 140 rigid and non-rigid prostheses [30]. The other two framework fractures were reported in a multi-center retrospective study. In this study, the frameworks were most commonly made of a high noble alloy, some were made of titanium, and a few were made of gold. The framework fractures in this study involved the attachment parts of the prostheses [50].

### 4.2. Survival

#### 4.2.1. Overall Survival

In this review, overall prosthesis survival refers to any TIFP that remained functional in the mouth over the length of the study period, despite any minor complications. Minor complications encountered included: veneer fracture that did not require removal of the prosthesis, screw loosening, cement failure and peri-implant/periodontal bone loss not detrimental to the survival of the prosthesis. In one study, complications were more severe, with implants and/or teeth needing to be extracted. However, in this study, the prostheses could be recemented without needing to be remade . Overall 502 out of 531 TIFPs remained functional in 18 studies that had the data available for review. The main cause of prosthesis failure was loss of the natural abutment due to fracture, caries or periapical pathology (*n* = 13), or loss of the implant abutment (*n* = 8) resulting in insufficient prosthesis support. Other factors that influenced the overall survival included framework fracture (*n* = 4), tooth intrusion (*n* = 1), permanent debonding (*n* = 1), and unspecified technical complications (*n* = 2) as described in the study by Bragger *et al.* 2005 [51]. The studies reviewed included patients observed for 6 to 158 months. Six studies were not included in this section as it was unclear whether failing complications occurred in the same prosthesis or in different prostheses.

#### 4.2.2. Complication-Free Survival

Fifteen studies provided sufficient information regarding complication-free survival. Out of the studies reviewed 336 out of 439 prostheses showed no technical or biological complications and remained functional in the mouth. Ten studies were excluded from this aspect of the literature review due to insufficient information on treatment complications. Such studies often quoted figures according to the type of complication, making it unclear whether more than one complication occurred in the same prosthesis or if the complications were spread between a number of prostheses.

Most complications, which were or were not detrimental to prosthesis survival, were related to veneer fracture or wear (*n* = 26). This was followed by complications related to the natural abutment (*n* = 17) and implant related complications (*n* = 17). Intrusion occurred in 7 cases and retention related complications in 17 cases. Other complications included horizontal screw loosening or fracture (*n* = 3), necessary occlusal adjustments (*n* = 2) and other unspecified complications (*n* = 16). Eighteen cases of abutment fracture in nine patients in a study by Block *et al.* were excluded due to manufacture defect in the laser weld of the Omniloc abutment [31]. These abutments were eventually replaced with abutments lacking a laser weld and fracture did not re-occur.

## 5. Discussion

The aim of this review was to examine the literature and provide an overview of complications associated with tooth-to-implant-connected fixed partial dentures, as well as to provide information on overall prosthesis survival and complication-free survival. The quality of the evidence comprising this literature review varies. Although the texts included in this review dealt with both prospective and retrospective cohort studies, some incorporated control groups or cross-arch controls, and some studies also randomly assigned different treatments into groups. A number of studies reviewed clearly defined the parameters for data collection. However, in others, results were arbitrary and subject to investigator interpretation. A clear example of this was the measurement of peri-implant bone loss, which was measured using either clinical measurements, radiographs, or, in one study, implant threads, which made distance measurement subjective. This was also evident in a study by Block *et al.*, where study photographs were used to monitor intrusion [31], whereas, in other studies, only clinical observations were used. This lead to a large number of cases of intrusion detected when compared to other studies. In certain studies, it was also unclear who the investigator collecting the data was and whether he was biased. Despite the possible application of this method of treatment in a number of clinical situations, randomized control studies were uncommon.

This literature review found 25 studies that fit the inclusion criteria. Different methodologies and measured results meant that some texts were excluded from different aspects of this review. A more standardized approach with regard to data collection for dental, periodontal, implant, peri-implant as well as prosthesis conditions from investigators and study authors would simplify and improve interpretation of data for meta-analysis, especially considering the multifaceted nature of TIFP treatment. The different study methodologies and measurements collected meant that it was not possible to undertake a meta-analysis on the results of the papers available in the literature. However, it was possible to follow certain trends and draw relevant conclusions.

Complications of the natural abutments were the most common cause of failure. This was associated with the use of compromised teeth for prosthesis support, resulting in a number of natural abutments being extracted due to fracture or caries. In fact, studies that made use of predominately vital abutments over RCT teeth reported no tooth fractures [8,9,44,50]. This evidence is what may be expected and is similar to what has been documented for conventional FPDs. In this review, 1.2% of abutment teeth fractured, whereas 2.1% in a review of conventional FPDs [57]. Out of the 16 documented natural tooth fractures in this review, 6 were previously RCT, while the endodontic status of the other 10 fractured teeth was not specified.

Dental caries developed in a number of situations where loss of retention on the natural abutment went unnoticed due to retention of the prosthesis on the implant. This was evident in the study by Bragger *et al.*, where four carious lesions started following a loss of retention and eventually led to the same four cases, losing the related implants and corresponding prostheses. It is worth mentioning that in this study most of the lost natural abutments were RCT and restored with cast posts and cores [51]. Apart from the risk of caries, debonding of the prosthesis from the natural abutment led to the intrusion of teeth [30,31]. Intrusion was also evident when teeth were intentionally connected to implants in a non-rigid manner [34] or when two-piece rigid prostheses became non-rigid due to loosening/fracture of the attachment screw [50]. From this review, it is possible to say that, when implants were rigidly connected to teeth, intrusion was rarely reported, although it must be considered that this may be due to difficulty in actually visualizing and detecting intrusion in rigid cases. With this in mind, it may be advantageous to ensure that the tooth is permanently cemented to the prosthesis, despite possible concerns with prosthesis retrievability. From the observations in this review, it is impossible to deduce the etiology of intrusion.

Although limited data was available, no prostheses were lost due to periodontal complications resulting from connecting teeth to implants, and reported cases of periodontitis were uncommon. However, this may have been due to the fact that observing the periodontal condition was not a priority of the studies included in this review.

As described in Table 7, no single method of connecting teeth to TIFPs is ideal in all respects, and it is up to the clinician to best evaluate the clinical scenario and decide on the mode of connection that best suits the case.

Prosthesis design is a disputed aspect of TIFP treatment. Most controversy is centered on the rigidity of the superstructure. As previously described, the main reason behind this controversy is the mismatch in mobility between implants and teeth. This mismatch theoretically causes the tooth to act as a cantilever and the implant to bear most of the force whenever a rigid connection is employed [9]. The consequences of this may be marginal bone loss or technical complications related to the implant (Table 8). This has led some clinicians to non-rigidly connect implants and teeth. Despite the theoretical reasoning, the reviewed studies showed a negative trend when it came to non-rigid connection of teeth to implants. Apart from the increased evidence of dental intrusion when teeth were non-rigidly connected to implants [7,31,34], studies show more complications and more inter-review appointments needed to deal with technical complications [10,31,35,51]. In the study by Nickenig *et al.*, only 3 of 56 rigid TIFPs were affected by technical complications, while 8 of 28 non-rigid TIFPs needed modification [35]. Technical complications were also evident when attachments were used, which were locked to form a rigid prosthesis [42,50].

From this review, it was evident that prosthesis framework fractures were rare occurrences, with only seven fractures described. Two framework fractures occurred in the only study that used rigid Zirconia bridges [56], and the other five occurred in metal constructions, with two of these fractures occurring around prosthetic attachments [50]. In light of the limited evidence on Zirconia TIFPs, it may be recommended that this material is used with caution. As may be expected, veneer fracture was a more common complication. Although the number of patients treated with composite or acrylic bridges was limited, it was noted that more composite/acrylic fractures (*n* = 9/50) occurred than when porcelain veneering was used (*n* = 46/391). Fifteen fractures were not attributed to any material. As may be expected, there were also fewer complications related to wear and esthetics when porcelain veneering was used. In light of the evidence in this literature review, it may be advisable to utilize rigid metal frameworks veneered with porcelain in TIFP cases.

The studies included in this literature review also demonstrated positive outcomes for the implant abutment and peri-implant bone. Concerns about the bending forces on the prosthetic components and on the implant itself were not clinically manifested. This literature review found a low incidence of prosthetic screw fracture, as well as implant fracture, which was comparable to that found in literature reviews of FSFPs. In a literature review by Pjetursson *et al.* on implant- implant-supported FPDs, fracture of implants was a rare complication with a cumulative incidence of 0.4% (95% CI: 0.1–1.2%) after 5 years and 1.8% (95% CI: 1.2–2.6%) after 10 years [58]. In this literature review, implant fracture occurred in 7 of the 1610 implants reviewed (0.4%). Despite the positive outcomes for the implant abutments, a number of early implant loses (Table 4) may have been avoided by clinically testing the level of osseointegration before connecting the implant to a naturally mobile tooth abutment. The need to ascertain osseointegration may be more important in TIFP cases as opposed to FSFP cases were both abutments are rigid.

Marginal bone loss around fixtures, exceeding the parameters set by Albrektsson for implant success was rare [48], with some studies also demonstrating an overall increase in marginal bone levels [8,9]. In other studies, the level of fixture marginal bone loss was lower in TIFP cases than in FSFP cases [9,44,50]. This mismatch in marginal bone loss between FSFP cases and TIFP cases may be due to decreased occlusal forces on TIFP prosthesis as a result of proprioceptive feedback from the PDL of the natural abutment. Similarly, studies exploring marginal bone level changes in implants supporting cantilevered prostheses also demonstrate no significant differences in bone level in implants supporting prostheses without cantilevers [59,60,61]. However, prosthetic complications are increased in cantilevered prostheses [62]. To better understand the clinical relevance of tooth-to-implant prosthetic treatment, clinical studies should be carried out comparing this method of treatment to cantilevered implant prostheses, preferably in randomized cross-arch studies.

## 6. Conclusions

**Clinical implications:** Although based on a limited number of cohort studies, with small sample sizes, it can be concluded that connecting teeth to implants is a viable treatment option, as agreed on by the authors of the studies reviewed. A number of texts included in this study recommended rigid connection of the natural and fixture abutments in order to avoid intrusion of the natural abutment [8,9,11,40,44,51,52]. There was also a trend for investigators to restore using a metal framework veneered in porcelain [29,34,40,50].

A number of authors from the studies selected expressed concern about the reduced retrievability resulting from permanently cementing TIFPs to the natural abutment [8,29,31,47]. Despite this, it may be advisable to permanently cement a rigid one-piece casting to the natural abutment to avoid a number of possible technical complications. These complications include failure of temporary cement, fracture of attachments, loosening or fracture of attachment screws, and fracture of veneering material. Due to poor retrievability of permanently cemented one-piece prostheses, this treatment should be avoided in smokers or patients with less than optimal oral hygiene, as removal of the prosthesis for peri-implant treatment would be difficult. It is also advisable to avoid the inclusion of teeth with poor prognosis, mainly RCT teeth, to ensure a favorable long-term prognosis for TIFPs.

From this review, it is possible to conclude that rigid TIFPs, permanently secured to teeth with sufficient coronal structure, while restricting the use of attachments in patients with good oral hygiene and sound implant positioning offer a good long-term treatment option for patients where solely free-standing implant-supported options may not be possible.

**Further research:** Larger randomized control studies and other clinical studies with fewer variables are required. Studies comparing tooth-to-implant-connected treatment with other forms of treatment including implant-supported cantilevered prostheses are needed to better understand the place of TIFP treatment in oral rehabilitation. Further studies should present standardized results regarding all aspects of TIFP treatment.

## Figures and Tables

**Table 1:** PICO (Participants, Intervention, Comparison, and Outcome) Question used to focus literature search.

Participants	Partially Edentulous Patients Requiring Tooth-Implant Fixed Prostheses (TIPFs) Treatment
Interventions	Rigid/non-rigid tooth-to-implant connection.
	Permanent/temporary cement use for tooth cementation.
	Screw/cement retained for tooth/implant fixation.
	Different framework and veneering materials.
Comparison	Studies with similar interventions on tooth supported by FSFPs.
Outcomes	Complications, complication-free survival, and overall survival of different tooth-to-implant fixed prosthesis treatment modalities.

**Table 2:** Table of articles selected for review.

No.	Authors	Title	Source	Institute	Study Design	No. of Patients	Study Aim	Observation Period
1	Akca K, Cehreli MC.	Two-Year Prospective Follow-up of Implant/Tooth-Supported Versus Freestanding Implant-Supported Fixed Partial Dentures.	Int J Periodontics Restorative Dent. 2008, 28, 593–599	Faculty of Dentistry, Hacettepe, University, Ankara, Turkey	Prospective case study	29	To Compare prosthetic outcomes of rigidly connected short-span TIFPs and FSFPs.	24 months
2	Åstrand P, Borg K, Gunne J, Olsson M.	Combination of Natural Teeth and Osseointegrated Implants as Prosthesis Abutments: A 2-Year Longitudinal Study	Int J Oral Maxillofac Implants 1991, 6, 305–312.	Maxillofacial Unit at the University of Umeå	Prospective case study with cross-arch control; Randomized	23	To perform cross-arch comparisons between FSFPs and TIFPs.	24 months
3	J Gunne, Astrand P, Ahlen K, Borg K, Olsson M.	Implants in partially edentulou patients. A longitudinal study of bridges supported by both implants and natural teeth	Clin. Oral. Impl. Res. 1992, 3, 49–56	Maxillofacial Unit at the University of Umeå	Prospective case study with cross-arch control; Randomized	23	To compare the outcomes of FSFPs with TIFPs.	36 months
4	Olsson M, Gunne J, Astrand P, Borg K.	Bridges supported by free-standing implants *versus* bridges supported by tooth and implant	Clin. Oral. Impl. Res. 1995, 6, 114–121	Maxillofacial Unit at the University of Umeå	Prospective case study with cross-arch control; Randomized	23	To evaluate the same bridges as those presented in 1991 after 5 years in function.	60 months
5	Gunne F, Ástrand P, Lindh T, Borg K, Olsson M.	Tooth-Implant and Implant Supported Fixed Partial Dentures: A 10-Year Report	Int J Prosthodont 1999, 12, 216–221.	Maxillofacial Unit at the University of Umeå	Prospective case study with cross-arch control; Randomized	23–20 attending at 10 years	To compare FSFPs with TIFPs after 10 years of function.	120 months
6	Block MS, Lirette D, Gardiner D, Li L, Finger IM, *et al.*	Prospective Evaluation of Implants Connected to Teeth.	Int J Oral Maxillofac Implants. 2002, 17, 473–487	Authors affiliated with the Department of Oral and Maxillofacial Surgery, Louisiana State University	Prospective case study with cross-arch control; Randomized	40	To examine the effect on teeth and implants when rigidly or non-rigidly connected in a cross-arch study.	60 months
7	Bragger U, Karoussis I, Persson R, Pjetursson B, Salvi G, *et al.*	Technical and biological complications/failures with single crowns and fixed partial dentures on implants: a 10-year prospective cohort study.	Clin. Oral Impl. Res. 2005, 16, 326–334	Clinic for Periodontology and Fixed Prosthodontics University of Bern	Prospective case study	21	To assess the incidences of technical and/or biological complications and failures occurring in partially edentulous patients with fixed reconstructions on implants over 10 years.	120 months
8	Cordaro L, Ercoli C, Rossini C, Torsello F, Feng C.	Retrospective evaluation of complete-arch fixed partial dentures connecting teeth and implant abutments in patients with normal and reduced periodontal support.	J Prosthet Dent 2005, 94, 313–320	Authors affiliated with the Eastman Dental Hospital, Rome, Italy; Eastman Dental Center Rochester, NY.	Retrospective case study	20	The aim of this retrospective study was to evaluate the clinical outcomes of complete-arch TIFPs in patients demonstrating normal or reduced periodontal support.	24–94 months average of 36.5 months
9	Ericsson I, Lekholm U, Branemark PI, Lindhe J, Glantz PO, *et al.*	A clinical evaluation of fixed-bridge restorations supported by the combination of teeth and osseointegrated titanium implants.	J Clin Periodontol 1986, 13, 307–312	Department of Periodontology, University of Gothenburg,	Prospective case study	10	To investigate whether titanium fixtures could be used as combined abutments with teeth in fixed bridgework.	6–30 months average of 17.4 months
10	Heinemann F, Mundt T, Biffar R.	Retrospective evaluation of temporary cemented, tooth and implant supported fixed partial dentures	Journal of Cranio-Maxillofacial Surgery 2006, 34, Suppl. 2, 86–90	Private practice in Germany.	Retrospective case study	47	The aim of this study was to evaluate TIFPs, and determine which cement is best suited for temporary cementation.	48 months
11	Hosny M, Duyck J, van Steenberghe D, Naert I.	Within-Subject Comparison Between Connected and Non connected TIFP: Up to 14-Year Follow-up Study	Int J Prosthodont. 2000, 13, 340–346.	University Hospitals of Catholic, University of Leuven, Belgium	Retrospective, case study with cross-arch control	18	This long-term follow-up study aimed to compare the outcome of TIFPs and FSFPs.	168 months
12	Kindberg H, Gunne J, Kronström M.	Tooth- and Implant-Supported Prostheses: A Retrospective Clinical Follow-up up to 8 Years	Int J Prosthodont 2001, 14, 575–581	Department of Prosthetic Dentistry, Central Hospital, Skövde, Sweden	Retrospective case study	36	To evaluate clinical treatment outcomes of fixed prostheses in different sizes and with combinations of different numbers of teeth and implants as abutments	14 months to 106 months
13	KoczorowskiR, Surdacka A.	Evaluation of bone loss at single-stage and two-stage implant abutments of fixed partial dentures	Adv Med Sci. 2006, 51 Suppl. 1, 43–45.	Authors affiliated with the University of Medical Sciences in Poznan	Prospective case study	32	To evaluate alveolar bone loss at single-stage and two-stage implants as abutments of fixed partial dentures used to replace missing teeth.	76 implants reviewed at 24 months, 50 reviewed up to 72 months
14	Lindh T, Bäck T, Nyström E, Gunne J.	Implant *versus* tooth-implant supported prostheses in the posterior maxilla: a 2-year report	Clin. Oral Impl. Res. 2001, 12, 441–449.	Department of Prosthetic Dentistry at Umeå University.	Prospective clinical study with cross-arch control	26	To compare the biological and mechanical consequences of implants placed in the posterior maxilla connected to teeth, or when used in FSFPs.	24 months
15	Lindh T, Dahlgren S, Gunnarsson K, Josefsson T, Nilson H, *et al.*	Tooth-Implant Supported Fixed Prostheses: A Retrospective Multicenter Study	Int J Prosthodont 2001, 14, 321–328.	Multi-centre (6 centres)	Retrospective case study	111	To investigate the implant survival rate and loss of marginal bone, as well as indications and complications pertinent to TIFP treatment.	36 months
16	Mundt T, Hinemann F, Schankath C, Schwahn C, Biffar R.	Retrospective and clinical evaluation of retrievable, tooth-implant supported zirconia-ceramic restorations	Acta Odontol Scand 2013, 71(5), 1326–1334	Private practice, Germany	Retrospective case study	23	To assess retrievable TIFPs made of veneered zirconia cores as a viable treatment option.	12.7–47.9 average of 28.8 months
17	Naert I, Quirynen M, Van Steenberghe D, Darius P.	A six-year prosthodontic study of 509 consecutively inserted implants for the treatment of partial edentulism.	J Prosthet Dent 1992, 67, 236–245.	Leuven University Clinic.	Retrospective case study	146 (80 tooth-to-implant bridges)	To investigate the connection between teeth and implants and its possible harmful effects on fixture loss and bone loss as well as to investigate the use of composites or porcelain on the occlusal surfaces of TIFPs.	2 to 77 months
18	Naert I, Duyck J, Hosny M, van Steenberghe D.	Freestanding and tooth-implant connected prostheses in the treatment of partially edentulous patients Part I: An up to 15-years clinical evaluation.	Clin. Oral Impl. Res. 2001, 12, 237–244	Dept. of Periodontology and of Prosthetic Dentistry at the Hospitals of the Catholic University of Leuven.	Retrospective case study with cross-arch control	123	To compare TIFP and FSFP treatment modalities with each other based on implant, tooth and prosthesis complications.	18–180 months
19	Nickenig HJ, Schafer C, Spiekermann H.	Survival and complication rates of combined tooth–implant-supported fixed partial dentures	Clin. Oral Impl. Res. 2006, 17, 506–511	Based on the treatment documentations of a Bundeswehr dental clinic (Cologne-Wahn German Air Force Garrison)	Retrospective case study	83 patients	To review the incidence of biological and technical complications in case of TIFP treatment on the basis of survival data regarding clinical cases.	26.4–99.6 average of 56.8 months.
20	Noda K, Arakawa H, Maekawa K, Hara ES, Yamazaki S.	Identification of risk factors for fracture of veneering materials and screw loosening of implant-supported fixed partial dentures in partially edentulous cases	Journal of Oral Rehabilitation 2013, 40, 214–220	Fixed Prosthodontic Clinic of Okayama University Dental Hospital, Okayama, Japan	Retrospective case study	120 for veneer fracture, 81 for abutment screw loosening.	To identify the risk factors for fracture of veneering materials and screw loosening of implant-supported fixed partial dentures in partially edentulous cases.	Average of 48 months for screw loosening group and 30 months for veneer fracture group.
21	Özkan Y, Akoğlu B, Kulak-Özkan Y.	Five-year Treatment Outcomes with Four Types of Implants in the Posterior Maxilla and Mandible in Partially Edentulous Patients: A Retrospective Study	Int J Oral Maxillofac Implants 2011, 26, 639–647	University of Marmara, Department of Oral Surgery and Depart-ment of Prosthetic Dentistry, Istanbul, Turkey,	Retrospective case study	83	To evaluate the clinical and radiologic outcomes of four types of implants and their suprastructures in the posterior maxilla and mandible in partially edentulous patients after 5 years of functional loading.	60 months
22	Palmer RM, Howe LC, Palmer PJ.	A prospective 3-year study of fixed bridges linking Astra Tech ST implants to natural teeth	Clin. Oral Impl. Res. 2005, 16, 302–307	Authors affiliation: Departments of Perio. and Prosth. GKT Dental Institute, King’s College, London.	Prospective case study	19	To assess the clinical and radiographic performance of the teeth and implants used to support three-unit fixed bridges subjected to normal functional loads.	36 months
23	Rammelsberg P, Schwarz S, Schroeder C, Bermejo J, Gabbert O.	Short-term complications of implant-supported and combined tooth-implant-supported fixed dental prostheses	Clin. Oral Impl. Res. 2013, 24, 758–762	Department of Prosthodontics at the University Hospital of Heidelberg.	Retrospective case study	132	To investigate the complications of metal-ceramic and all-ceramic FDPs supported by implants or by a combination of teeth and implants.	Average of 28 months.
24	Romeo E, Lops D, Margutti E, Ghisolfi M, Chiapasco M, *et al.*	Long-term Survival and Success of Oral Implants in the Treatment of Full and Partial Arches: A 7-year Prospective Study with the ITI Dental Implant System	Int J Oral Maxillofac Implants 2004, 19, 247–259	Dental Clinic, Department of Medicine Surgery and Medicine, University of Milan, Italy.	Prospective case study	201	To evaluate the medium- to long-term survival and success of different implant-supported prostheses supported by ITI implants and to determine whether significant differences in survival and success could be observed for different implant placement sites.	46.2 months
25	Tangerud T, Grønningsæter AG, Taylor A.	Fixed Partial Dentures Supported by Natural Teeth and Brånemark System Implants: A 3-year Report	Int J Oral Maxillofac Implants 2002, 17, 212–219	Dental School, University of Bergen, Norway	Prospective case study	30	To evaluate TIFPs in a variety of clinical situations.	36 months

Note: Studies 2, 3, 4, and 5 followed the same patient cohort.

**Table 3:** Table of studies detailing failures of implants supporting TIFPs.

Author/Year	Methodology	Implant Brand	Number of Connected Implants	Connected Implants Lost	Peri-Implant Bone Changes	Observation Period
**(Akca and Cehreli 2008)**	Prospective study comparing TIFPs and FSFPs.	ITI/Straumann	34	0	+0.19 mm (±0.52 mm) change in bone level which was significantly less bone loss than in FSFPs.	24–30 months (mean 26 months)
**(Astrand *et al.* 1991, Gunne *et al.* 1992, Olsson *et al.* 1995, Gunne *et al.* 1999)**	Prospective study comparing TIFPs and FSFPs, randomized cross-arch control studt.	Nobel Biocare	23	2	−0.5–0.7 mm over 10 years not statistically significant to the contralateral side supporting FSFP implants.	120 months
**(Block *et al.* 2002)**	Prospective study comparing rigid and non-rigid TIFPs, randomized cross-arch study.	Omniloc	60	1	0.91 mm Average bone loss. No significant difference in bone loss around rigidly and non-rigidly connected implants. Four implants developed bone loss >2 mm.	60 months
**(Bragger *et al.* 2005)**	Prospective study following single crown, FSFP and TIFP restorations.	ITI/Straumann	22	3	Three implants lost due to excessive bone loss.	120 months
**(Cordaro *et al.* 2005)**	Retrospective study analyzing the performance of full-arch TIFPs.	3i Implant and ITI/Straumann	90	1	87 implants had stable bone levels during the observation period while 3 had bone loss than >2 mm.	24–94 months (mean 36.5 months)
**(Ericsson *et al.* 1986)**	Prospective study following implants rigidly and non-rigidly connected to teeth.	Branemark	41	0	<1 mm marginal bone loss round most implants; 1–3 mm bone loss around 3 fixtures and >3 mm bone loss around2 implants in one patient and with rigid connection.	6–30 months (mean 17.4 months)
**(Heinemann *et al.* 2006)**	Retrospective evaluation of different temporary cements in TIFP cases.	Tiolox	155	1	Two implants developed peri-implantitis of which one was lost.	48 months
**(Hosny *et al.* 2000)**	Retrospective study of TIFPs with cross-arch FSFP control.	Branemark	30	0	1.9 mm: Average bone loss over 15 years (2.2 mm/year for the first 6 months, 0.015 mm/year thereafter); More bone loss in FSFP group; however, difference not significant.	15–168 months (mean 78 months)
**(Kindberg *et al.* 2001)**	Retrospective analysis of implants rigidly or non-rigidly connected to teeth.	Nobel Biocare	112	6	After 1 year, 46 implants showed marginal bone loss, 31 up to one thread, 11 up to two threads, 2 up to three threads and 2 up to four threads, for the three and five year examination progression of bone loss was minimal.	14–107 months (mean 58.3 months)
**(Koczorowski and Surdacka 2006)**	Prospective evaluation of posterior implants connected to teeth.	Osteoplant	76	0	−0.70 mm ±0.50 after two years and −1.73 mm ±0.41 after six years of mean marginal bone loss.	24–72 months (mean 43 months)
**(Lindh *et al.* 2001a)**	Prospective study of TIFPs with cross-arch control FSFPs in Kennedy Class I patients.	Nobel Biocare	26	1	−0.09 ± 0.52 mm around the posterior connected implant. The difference in bone loss from loading to 24 months was significant for posterior implant in FSFPs but not significant for posterior implants in TISP.	24 months
**(Lindh *et al.* 2001b)**	Multi-centre retrospective study following TIFPs.	Nobel Biocare and Straumann	185	5	1.7 mm, SD 0.8 mm bone loss at 12 months in 9 of the 74 implants reviewed for the whole 3 years, 0.3 mm, SD 0.7 many more bone loss in the other 65 implants. The subsequent loss of marginal bone during the second and third years for these two groups of implants was lower (*n* = 9: 0.3 mm, SD 0.9 mm; *n* = 65: 0.1 mm, SD 0.5 many more).	36 months
**(Mundt *et al.* 2013)**	Retrospective study assessing zirconia TIFPs.	38 Tiolox, 8 Ankylos, 5 Straumann	51	0	No marginal bone loss measurements however, by the end of the examination period only one implant had bleeding on probing.	12.7–47.9 months (mean: 28.8 months)
**(Naert *et al.* 1992)**	Retrospective analysis of FSFPs and TIFPs.	Branemark	80	5	1.02 mm mean bone loss in Year 1 followed by 0.10 mm bone gain in year two. No statistical difference between bone loss around the ditstal implant in connected and non-connected cases.	72 months
**(Naert *et al.* 2001)**	Retrospective analysis of TIFPs compared to a control group of similar FSFPs.	Branemark	339	10	No statistical difference from 0 to 6 months between FS, non-rigid and rigid group, there was no statistical difference in bone loss from 6 to 180 years between the FSFP group 0.02 mm a year and non-rigid group 0.04 mm a year. There was, however, a statistical significant difference between the rigid group 0.09 mm a year and the FSFP group (*p* = 0.004).	18–180 months (mean 78 months)
**(Nickenig *et al.* 2006)**	Retrospective analysis of implants rigidly and non-rigidly connected to teeth.	85% Branemark and Straumann; 15% including Replace, Friadent, Ankylos and others	142	0	5 mm probing depths were found in <1% of implants after 5 years.	26–100 months (mean 56.8 months)
**(Ozkan *et al.* 2011)**	A retrospective study of single implant crowns, TIFPs and FSFPs.	Straumann, swiss plus, camlog, Friadent.	9	0	All implants met the criteria for success. All implants were surrounded by stable healthy tissue with crestal bone level changes not significantly different between TIFP and FSFP implants.	60 months
**(Palmer *et al.* 2005)**	A prospective study of rigidly connected teeth and implants via short-span bridges in Kennedy Class II cases	Astra tech	19	0	Up to 1.2 mm of bone loss seen in 9 patients; 10 patients experienced no change or an increase in bone level around connected implants.	36 months
**(Romeo *et al.* 2004)**	A prospective study of various types of implant-supported prostheses designs.	ITI	31	3	N/A	16–84 months (mean 46.2 months)
**(Tangerud *et al.* 2002)**	Prospective study monitoring 30 rigid TIFPs.	Branemark	85	2	Bone loss of 0.8 mm ± 1.1 mm around connected implants from time of loading to 3 years review.	36 months

**Table 4:** Table of failed implants.

Author/Year	Location of Failed Implant	Opposing Dentition	Connection Type	Reconstruction Length	FPD Retention	Time after Loading	Implant Brand	Reason for Failure
**(Astrand *et al.*, 1991, Gunne *et al.*, 1992, Olsson *et al.*, 1995, Gunne *et al.*, 1999)**	Posterior mandible	Complete Removable	Rigid	3-unit	Screw	Within 18 months	Nobel Biocare	Loss of integration
	Posterior mandible	Complete Removable	Rigid	3-unit	Screw	Within 18 months	Nobel Biocare	Loss of integration
**(Block *et al.* 2002)**	Posterior mandible	Complete Removable	Rigid	3-unit	Screw	36 months	Omniloc	Loss of integration without inflammation
**(Bragger *et al.* 2005)**	N/A	Fixed	Rigid	N/A	N/A	Within 60 months	ITI/Straumann	Primary biological complication
	N/A	Fixed	Rigid	N/A	N/A	Within 60 months	ITI/Straumann	Bony defect followed by fracture
	N/A	Fixed	Rigid	N/A	N/A	Within 120 months	ITI/Straumann	Loss of integration
**(Cordaro *et al.* 2005)**	Posterior Maxilla	Combined fixed removable prosthesis	Non rigid	12-unit	Permanent Cement	7 months	Straumann	Mobility
	N/A	N/A	Rigid	N/A	Temporary Cement	N/A	Tiolox	Peri-implant disease
**(Kindberg *et al.* 2001)**	Maxilla	N/A	Non rigid	12-unit	Screw	36 months	Nobel Biocare	Loss of integration
	Maxilla	N/A	N/A	N/A	Screw	36 months	Nobel Biocare	Loss of integration
	Maxilla	N/A	N/A	N/A	Screw	36 months	Nobel Biocare	Loss of integration
	Maxilla	N/A	Rigid	10-unit	Screw	60 months	Nobel Biocare	Loss of integration
	Maxilla	N/A	N/A	N/A	Screw	60 months	Nobel Biocare	Loss of integration
	Maxilla	N/A	N/A	N/A	Screw	60 months	Nobel Biocare	Loss of integration
**(Lindh *et al.* 2001a)**	Posterior Maxilla	N/A	Rigid	Unilateral short span	Screw	Within 3 months	Nobel Biocare	Mobility
**(Lindh *et al.* 2001b)**	Posterior Maxilla	N/A	N/A	N/A	N/A	12 months	Straumann or Nobel Biocare	Loss of integration
	Posterior Maxilla	N/A	N/A	N/A	N/A	12 months	Straumann or Nobel Biocare	Loss of integration
	Maxilla	N/A	N/A	N/A	N/A	12 months	Straumann or Nobel Biocare	Loss of integration
	Maxilla	N/A	N/A	N/A	N/A	12 months	Straumann or Nobel Biocare	Loss of integration
	Maxilla	N/A	N/A	N/A	N/A	N/A	Straumann or Nobel Biocare	Loss of integration
**(Naert *et al.*, 1992)**	Posterior maxilla	N/A	Rigid	Unilateral short span	N/A	Within 36 months	Branemark	Fracture
	Posterior maxilla	N/A	Rigid	Unilateral short span	N/A	Within 36 months	Branemark	Fracture
	Posterior	N/A	Rigid	Unilateral short span	N/A	Within 22 months	Branemark	Loss of integration
	Posterior	N/A	Rigid	Unilateral short span	N/A	Within 22 months	Branemark	Loss of integration
	Posterior	N/A	Rigid	Unilateral short span	N/A	Within 22 months	Branemark	Loss of integration
**(Naert *et al.* 2001)**	N/A	N/A	Rigid	N/A	Screw	25–36 months	Branemark	Mobility
	N/A	N/A	Rigid	N/A	Screw	25–36 months	Branemark	Mobility
	N/A	N/A	Rigid	N/A	Screw	25–36 months	Branemark	Mobility
	N/A	N/A	Rigid	N/A	Screw	25–36 months	Branemark	Mobility
	N/A	N/A	Non-rigid	N/A	Screw	49–60 months	Branemark	Mobility
	N/A	N/A	Non-rigid	N/A	Screw	49–60 months	Branemark	Mobility
	N/A	N/A	Rigid	N/A	Screw	61–72 months	Branemark	Fracture
	N/A	N/A	Rigid	N/A	Screw	85–96 months	Branemark	Fracture
	N/A	N/A	Rigid	N/A	Screw	85–96 months	Branemark	Fracture
	N/A	N/A	Non-rigid	N/A	Screw	85–96 months	Branemark	Fracture
**(Romeo *et al.* 2004)**	Mandible	N/A	N/A	N/A	N/A	72–84 months	ITI/Straumann	Peri-implant disease
	Maxilla	N/A	N/A	N/A	N/A	36–48 months	ITI/Straumann	Peri-implant disease
	Maxilla	N/A	N/A	N/A	N/A	48–60 months	ITI/Straumann	Peri-implant disease
**(Tangerud *et al.* 2002)**	Maxilla	N/A	Rigid	N/A	Screw	12–24 months	Branemark	Mobility
	Maxilla	N/A	Rigid	N/A	Screw	24–36 months	Branemark	Peri-implant bone loss

**Table 5:** Table of studies detailing failures of natural teeth supporting TIFPs.

Author/Year	Methodology	No. of Connected Teeth	Initial Periodontal Condition	Initial Endodontic Condition	Method of Fixation	Lost to Dental Caries	Lost to Periapical Pathology	Lost to Tooth Fracture	Periodontal Bone Changes	Tooth Intrusion	Observation Period
**(Akca and Cehreli 2008)**	Prospective study comparing TIFPs and FSFPs.	34	crown root ratio 2:3.	4 RCT, 30 Vital	Permanent cement	0	0	0	N/A	0	24–30 months (mean 26 months)
**(Astrand *et al.* 1991, Gunne *et al.* 1992, Olsson *et al.* 1995, Gunne *et al.* 1999)**	Prospective study comparing TIFPs and FSFPs, randomized cross-arch control.	23	Healthy periodontium.	0 RCT, 23 Vital	Permanent cement	1		0	Not significantly different to control; 1 tooth developed mobility	0	120 months
**(Block *et al.* 2002)**	Prospective study comparing rigid and non-rigid TIFPs.	60	Healthy periodontium Crown root ration of at least 1:2.	27 RCT, 33 Vital	Permanent cement for non-rigid. Temporary cement for rigid.	0	0	2 rigid side 3 non rigid side(all RCT)	No significant bone loss	21 non rigid cases 14 rigid cases.	60 months
**(Bragger *et al.* 2005)**	Prospective study following SC, FSFP and TIFP restorations.	24	Supportive periodontal care given.	RCT and vital	Permanent cement	4	0	0	N/A	0	120 months
**(Cordaro *et al.* 2005)**	Retrospective study of TIFPs on teeth with normal and reduced periodontal support.	72	10 patients had >2/3 residual periodontium 10 patients <2/3 residual periodontium	N/A	Permanent cement for non-rigid. Temporary cement for rigid.	0	0	0	N/A	4 non-rigid cases with >2/3 periodontal support	24–94 months (mean 36.5 months)
**(Ericsson *et al.* 1986)**	Prospective study following implants rigidly and non-rigidly connected to teeth.	29	Supportive periodontal care given	N/A	N/A	0	0	0	No loss of alveolar bone around teeth.	1 non-rigid case	6–30 months (mean 17.4 months)
**(Heinemann *et al.* 2006)**	Retrospective evaluation of different temporary cements in TIFP cases.	108	N/A	N/A	Temporary or semi-permanent cement.	0	2		N/A	0	48 months
**(Hosny *et al.* 2000)**	Retrospective study of TIFPs with cross-arch FSFP control.	30	N/A	N/A	16 Temporary cement, 14 Permanent cement	0	0	0	N/A	0	15–168 months (mean 78 months)
**(Kindberg *et al.* 2001)**	Retrospective study of implants connected rigidly or non-rigidly to teeth.	85	Healthy periodontium	N/A	45 screw locked, cemented or telescopic	0	5		No significant bone loss	1 non-rigid case and 2 in rigid cases with non locked telescopic crowns.	14–107 months (mean 58.3 months)
**(Lindh *et al.* 2001a)**	26 TIFPs with cross-arch control FSFPs in Kennedy Class I patients.	26	16 intact periodontia; 10 <1/4 bone loss	15 RCT, 11 Vital	Temporary and Permanent cement	0	0	1	No increased mobility reported	0	24 months
**(Lindh *et al.* 2001b)**	Multi-centre retrospective study following TIFP	220	21 lost >1/3 of their periodontal support	49 RCT, 171 Vital	N/A	0	0	0	N/A	11 all debonded from prosthesis	36 months
**(Mundt *et al.* 2013)**	Retrospective study assessing zirconia TIFPs.	40	No BOP <4 mm probing depth	RCT or Vital	Semi-permanent cement	0	0	0	3 teeth with BOP	0	12.7–47.9 months (mean: 28.8 months)
**(Naert *et al.* 2001)**	Retrospective analysis of TIFPs compared to a control group of FSFPs.	313	N/A	N/A	46 temporary cement, 94 permanent cement	3	11	2	N/A	19 all debonded from prosthesis	18–180 months (mean 78 months)
**(Nickenig *et al.* 2006)**	Retrospective analysis of teeth rigidly and non-rigidly connected to implants.	132	N/A	N/A	N/A	0	3	0	10 teeth required periodontal treatment	0	26.4–99.6 months (mean 56.76)
**(Palmer *et al.* 2005)**	Rigidly connected teeth and implants via 3-unit bridges in Kennedy Class II cases.	19	Healthy periodontium	Vital	Temporary cement	0	0	0	No significant bone loss	0	36 months
**(Tangerud *et al.* 2002)**	Prospective study monitoring 30 rigid TIFPs.	86	N/A	40 RCT, 46 Vital	Cemented	0	0	1 RCT	Bone reduction of 0.1 mm ± 0.8 mm around teeth	0	36 months

**Table 6:** Table of studies detailing prosthetic failures.

Author/Year	No. of Tooth-Implant Prostheses		Prostheses Length	Prostheses Construction	Method of Fixation		Veneer Fracture	Framework Fracture	CEMENT FAILURE	Implant Screw Fracture/Loosening	Opposing Dentition	Observation Period
	Rigid	Non-Rigid			Tooth	Implant						
**(Akca and Cehreli 2008)**	34	0	3-unit	Metal-ceramic	Permanent cement	Permanent cement	0	0	0	0	N/A	24–30 months (mean 26 months)
**(Astrand *et al.* 1991, Gunne *et al.* 1992, Olsson *et al.* 1995, Gunne *et al.* 1999)**	23	0	3-unit	Metal-acrylic	Permanent cement	Screw	0	0	0	3	Removable	120 months
**(Bragger *et al.* 2005)**	22	0	10 3-unit; 6 4-unit; 4 5-unit; 2 10-unit;	Metal-ceramic	Permanent cement	10 cement 12 screw	2	0	4	4	Fixed	120 months
**(Cordaro *et al.* 2005)**	6	13	10–14-unit	15 Metal-ceramic; 4 Metal-composite	Permanent/Temporary cement	12 Permanent/Temporary cement; 7 screw	1 (composite)	0	N/A	0	14 Fixed; 1 Removable; 4 Mixed	24–94 months (mean 36.5 months)
**(Heinemann *et al.* 2006)**	65	0	Most four abutments; 12 2–3 abutments; 13 >4 abutments	Metal-ceramic	Semi-permanent/ Temporary cement	Semi-permanent/ Temporary cement	5	0	80 with temp cement; 4 with semi-permanent cement	N/R	N/A	48 months
**(Hosny *et al.* 2000)**	14	4	6 3-unit; 6 4-unit; 2 5-unit; 3 6-unit; 1 8-unit	14 Metal-cerami; 4 Metal-acrylic	14 Permanent cement; 16 Temporary cement	Screw	0	0	0	0	N/A	15–168 months (mean 78 months)
**(Kindberg *et al.* 2001)**	40	1	11 3-unit; 5 4-unit; 25 >5-unit	20 Metal-ceramic 20 Metal-acrylic 1 Metal-composite	45 screw locked copings. Cemented and Telescopic	Screw	4	0	0	1	N/A	14 months to 106 months
**(Lindh *et al.* 2001a)**	26	0	Unilateral	Metal-ceramic	Permanent/Temporary cement	Screw	0	0	2 with temporary cement	1	N/A	24 months
**(Lindh *et al.* 2001b)**	122	16	Most 1 implant to 1 tooth	131 Metal-ceramic 7 Metal-acrylic	N/A	N/A	3	2(attachment fractures)	0	2 (and 2 abutment screws loosened)	119 Fixed; 19 Removable	36 months
**(Mundt *et al.* 2013)**	31	0	15 3-unit; 4 4-unit; 6 5-unit; 4 6-unit; 1 8-unit; 1 12-unit	Zirconia with ceramic veneer	Semi-permanent cement	Semi-permanent cement	10	2	2	1 abutment screw loosened.	Fixed	12.7–47.9 months (mean 28.8 months)
**(Naert *et al.* 2001)**	49	34	N/A	106 Metal-ceramic 34 Metal- acrylic	94 Permanent cement; 46 Temporary cement.	Screw	N/A	3	25	Loose screw not reported; despite 3 abutment screw fractures	N/A	18–180 months (mean 78 months)
	57											
**(Noda *et al.* 2013)**	136		N/A	Metal-ceramic	N/A	N/A	22	N/A	N/A	4 of 58 had screw loosening	N/A	Mean of 37.3 months.
**(Ozkan *et al.* 2011)**	9		N/A	Metal-ceramic	Permanent cement	Screw/Permanent cement	0	0	0	0	Fixed and Removable	60 months
**(Palmer *et al.* 2005)**	19	0	Unilateral	Metal-composite	Temporary cement	Temporary cement	8	0	8	N/R	Fixed	36 months
**(Rammelsberg *et al.*, 2013)**	48	0	Mostly 3–4-unit	Metal-ceramic	Permanent/Semi-permanent cement	Permanent/Semi-permanent cement	7	0	3	N/R	N/A	Average of 28 months.
**(Romeo *et al.* 2004)**	13		N/A	Metal-ceramic	N/A	N/A	0	0	0	0	N/A	46.2 months
**(Tangerud *et al.* 2002)**	30	0	3–13 units, mean 8.6	16 Metal-ceramic, 14 Metal-composite	Cement	Screw	8	0	0	0	N/A	36 months

**Table 7:** Advantages and disadvantages of methods to secure a TIFP to a natural abutment.

	Intrusion	Retrievability	Caries Risk	Technical Complications
Temporary Cement	Likely	Good	Moderate	Low
Permanent Cement	Unlikely	Poor	Low	Low
Temporarily Cemented telescopic crowns	Possible	Good	Low	Low
Uncemented telescopic crowns	Likely	Good	Low	Low
Screw retained on coping	Possible	Good	Low	High
Permanent cement with locked TIFP attachment	Possible	Good	Low	High

**Table 8:** Advantages and disadvantages of rigid and non-rigid constructions.

	Intrusion	Biological Complications	Technical Complications
Rigid construction	Unlikely	No difference	Low
Non-Rigid construction	Likely	No difference	High
